# Dose-Dependent Behavioral Response of the Mosquito *Aedes albopictus* to Floral Odorous Compounds

**DOI:** 10.1673/031.013.12701

**Published:** 2013-11-12

**Authors:** Huiling Hao, Jingcheng Sun, Jianqing Dai

**Affiliations:** 1 Naval Medical Research Institute, 880 Xiangyin Road, Shanghai, 200433, China; 2 Guang Dong Entomological Institute, 105 Xingang Road, Guangzhou, 510260, China

**Keywords:** attractant, host-seeking disruption, plant volatile, spatial repellent

## Abstract

The value of using plant volatiles as attractants for trapping and spatial repellents to protect hosts against mosquitoes has been widely recognized. The current study characterized behavioral responses of *Aedes albopictus* (Skuse) (Diptera: Culicidae) to different concentrations, ranging from 6 to 96%, of several common floral odorous compounds, including linalool, geraniol, citronellal, eugenol, anisaldehyde, and citral, using a wind tunnel olfactometer system. The results indicated that female mosquitoes reacted differently to different concentrations of the tested compounds, and the reactions also were different when those chemicals were tested alone or in the presence of human host odor. When tested alone, anisaldehyde was attractive at all tested concentrations, eugenol was attractive only at concentrations of 48–96%, while citronellal, linalool, citral, and geraniol were attractive at lower concentrations and repellent at higher concentrations. When tested in the presence of a human host, all compounds except for anisaldehyde at all tested concentrations showed host-seeking inhibition to certain degrees. Based on the results, it was concluded that anisaldehyde was effective in attracting *Ae. albopictus* when used alone but could also remarkably inhibit the host-seeking ability at a concentration of 96%, while citral, geraniol, linalool, citronellal, and eugenol are suitable as spatial repellents.

## Introduction

Among the strategies of mosquito control, the use of plant volatiles or other attractive substances in attractant-baited traps or as spatial repellent agents has much attention (Kline and Lemiro 1998; Kline et al. 2003). Attractantbaited traps are intended to reduce mosquito population, while using spatial repellent agents are intended to interfere with a mosquito's host-seeking ability, thus reducing potential contact with humans and other animal hosts (Nolen et al. 2002). The combination of these two approaches is considered the latest technology in mosquito management (Nolen et al. 2002; Kline et al. 2003) and could achieve greater success than traditional approaches such as area-wide application of insecticides.

Numerous studies on identification of attractants and/or repellents of mosquitoes have been reported. For example, working with a wind tunnel, Jepson (1988) reported that *Aedes aegypti* was attracted to the odors of oxeye daisy, *Leucanthemum vulgare*, a common flowering plant. Further study revealed that the main component of the floral odors attracting mosquitoes was either a cyclic or a bicyclic monoterpene (Healy and Jepson 1988). *Culex pipiens* is attracted to plant essential oils and various substances, including sesquiterpenes, farnesol, green plant volatiles, and fatty acids (Bowen 1992). More recently, Jhumer et al. (2007) reported that 14 compounds from the flower odor of *Silene otites*, including benzaldehyde, eugenol, and linalool, were attractive to both *Culex pipiens molestus* and *Aedes aegypti*. On the other hand, many studies have been devoted to the evaluation of plant based substances, i.e., essential oils and their constituents, in searching for effective spatial repellents and/or alternatives to synthetic repellents (Barnard 1999; Dogan and Rossignol 1999; Tawatsin et al. 2001; Kline et al 2003; Trongtokit et al 2005; Amer and Melhorn 2006; Hao et al. 2006; Kang et al. 2009). As a result, a multitude of natural compounds have been identified as repellents of various mosquito species.

One of the research goals of our laboratory is to establish and optimize mosquito management tactics using combinations of attractantbaited traps and spatial repellents. We have developed an olfactometer (Hao et al. 2006) and have screened a large number of plantbased compounds. In the process, we noticed that for many plant volatiles it is difficult to simply classify them as attractants or repellents. The behavioral response of mosquitoes seems dependent on the dosage (amount present) of the tested samples. This phenomenon certainly complicates any attempt to formulate attractant-baited traps and spatial repellents. It is unfortunate that the effect of concentration amount has received very little attention in the existing literature. Using the olfactometer, the current study was set to characterize dosage effect on mosquito behavioral response, using *Aedes albopictus* (Skuse) (Diptera: Culicidae) as a model species, in an attempt to investigate new perspectives about attractants and spatial repellents and their applications in mosquito management. The compounds selected for this study include linalool, geraniol, citronellal, eugenol, anisaldehyde, and citral. All of them have been reported to have biological activities toward mosquitoes, and some of them are already formulated in spatial repellents as active constituents in spatial repellents (Ngoh et al. 1998; Oyedele et al. 2002; Barnard and Xue 2004; Hao et al. 2008).

**Figure 1.**
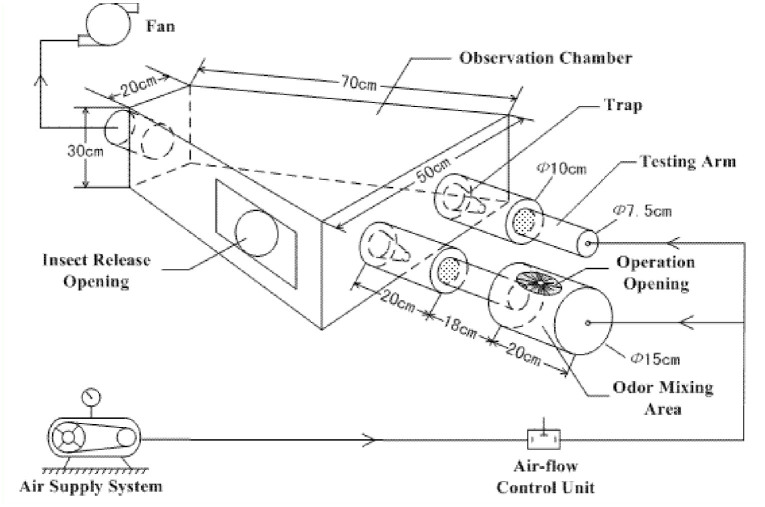
Schematic structure of olfactometer. High quality figures are available online.

## Materials and Methods

### Insect

*Aedes albopictus* (Shanghai strain) used in this study were originally collected from the metropolitan area of Shanghai, China, and were reared in the laboratory at 28 ± 1° C and 70 ± 5% RH under a 14:10 L:D photoperiod for 5 years. *Mus musculus* L. (Rodentia: Muridae) blood was fed to female adults. All experiments were conducted using bloodhungry 5–8 day-old adult females.

### Chemicals

Linalool (98%, CAS-No.78-70-6), geraniol (96%, CAS-No.106-24-1), citronellal (96%, CAS-No.106-23-0), citral (97%, CASNo. 5392-40-5), eugenol (99%, CAS-No.97- 53-0), anisaldehyde (99%, CAS-No.123-11- 5), and methanol (99.5%) were purchased from Xiyu Technology Company (www.ehsy.com).

### Testing apparatus and protocol

The testing apparatus, shown in Figure 1, was used to evaluate the behavioral response of *Ae. albopictus* females to different stimuli. Two testing ports with traps were positioned at one end of the observation chamber. A flow of charcoal filtered air at the rate of 0.2 m/s was directed into the observation chamber at both ports, and the airflow was measured by an airflow meter (model YF5000, Guang Zhou YI YI Automation Instrumentation Co., http://gzyy020.testmart.cn) before it was released into the olfactometer chamber. An exhaust fan was mounted at the other end of the observation chamber to facilitate the airflow.

To characterize behavioral responses, 25 mosquitoes were released into the observation chamber and allowed to acclimatize for 30 min to the air circulation. A testing compound sample (undiluted compounds and solutions of different concentrations) was introduced at one of the ports (treatment port) using a 2 × 2 cm filter paper soaked with 10 µL of the sample. A piece of filter paper soaked with 10 µL of methanol (solvent) was placed at the other port as a control (control port). The airflows diverted at the ports help distribute chemical molecules into the observation chamber. The numbers of mosquitoes collected in the traps at both ports were recorded 8 min after introduction of testing compound samples. All compounds were tested at the concentrations of 6, 12, 24, 48, and 96% in methanol. Solvent methanol also was tested.

To characterize host-seeking behavior, compounds were introduced in the presence of a human host (arms from one person) at the testing port and control port. Four human volunteers (two females and two males) were used in four replicated trials. No alcoholic drinks, tea, or coffee were allowed before or during testing, and the use of any fragrantcontaining product was banned as well. After each test, the arms were switched between the two testing ports. When testing compounds were changed, the apparatus was cleaned thoroughly and blank tests were performed to confirm that no more than 1 mosquito was trapped within 8 min before performing tests again.

All tests were conducted under 25 ± 1° C, 70 ± 5% RH, and 4500 lux. There were four replications of each treatment.

Under the same testing conditions as described above, the speed of evaporation of each compound was determined by the weight difference of the soaked filter paper (2 × 2 cm, soaked with 10 µL undiluted compounds) before and after 8 min of exposure in the testing apparatus. There were four replications for each compound.

**Table 1.**
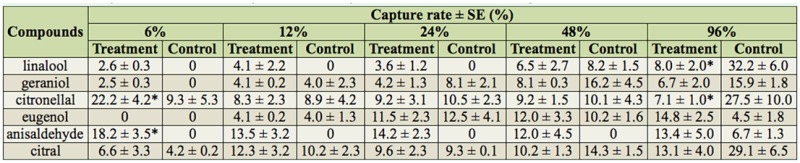
Behavioral response of *Aedes albopictus* to compounds at different dosages.

### Data analysis

Capture rate was calculated based on the number of mosquitoes in traps and the total number released. To quantify the attractive effects of compounds on *Ae. albopictus*, positive relative capture rate (%) was calculated as: (number of mosquitoes at treatment port - number of mosquitoes at control port)/total number of mosquitoes × 100. Thus, a positive relative capture rate indicates an attractive effect and negative values indicate a repellent effect. To quantify inhibition of compounds on *Ae. albopictus* response to human host scent, relative inhibition rate (%) was calculated as: (number of mosquitoes at control port - number of mosquitoes at treatment port)/total number of mosquitoes × 100. Thus, a positive relative inhibition rate indicates a repellent effect and negative values indicate an attractive effect. Speed of evaporation was analyzed by one-way ANOVA followed by Duncan's test for mean comparisons.

## Results

### Behavioral response to compounds

When compounds were administered alone, the results showed that the behavioral response (capture rates) were different among testing concentrations of a single compound and between different compounds (Table 1). For example, citronellal at 96% showed a significant repellent effect (capture rate 7.1% at treatment port vs. 27.5% at the control port), but at 6% it showed an attractant effect (capture rate 22.2% at treatment port vs. 9.3% at the control port). At 96%, a repellent effect was observed for linalool, citronellal, geraniol, and citral, but an attraction effect was observed for eugenol and anisaldehyde. Tests with methanol showed that it had no effect on mosquito response, as mosquitoes were not trapped in either port.

**Table 2.**
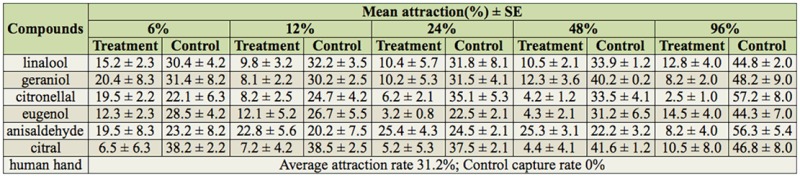
The host-seeking ability of *Aedes albopictus* when exposed to chemical compounds.

**Figure 2.**
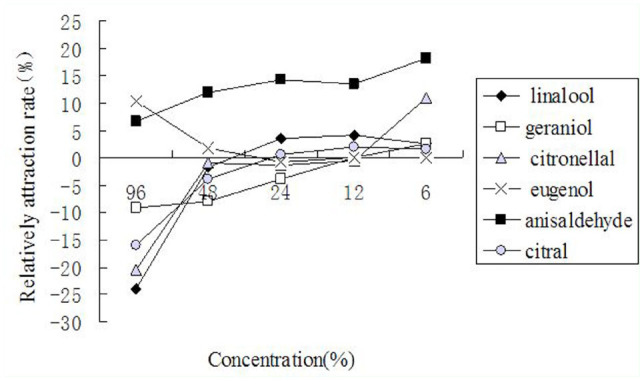
Attractive effect of compounds at the dosages of 96%, 48%, 24%, 12%, and 6% on *Aedes albopictus*. High quality figures are available online.

**Figure 3.**
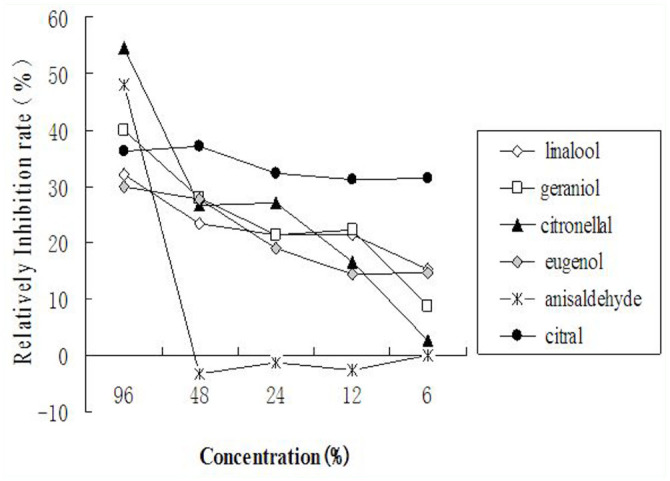
Inhibition effect of compounds at the dosages of 96%, 48%, 24%, 12%, and 6% on *Aedes albopictus* to host scent. High quality figures are available online.

Figure 2 shows the trends of relative capture rates of the compounds at different concentrations. Anisaldehyde and eugenol can be classified as attractants, as they were either attractive or had no effect at all at tested concentrations. Anisaldehyde at 6–48% showed a higher attractant effect (> 10% relative capture rates), while the attractant effect of eugenol diminished as the concentrations decreased. Dosage effect on classification of compounds was apparent. Citronellal, linalool, citral, and geraniol were attractants at lower concentrations and repellents at higher concentrations. Among them, citronellal had strong repellent effects at 96% (-20.4% relative capture rate) and strong attractant effects at 6% (12.0% relative capture rate). Linalool and citral at 96% were strong repellents (-24.0% and -16.1% relative capture rates, respectively), but at lower concentrations their effects on mosquito behavior were weak. Geraniol induced weak mosquito response regardless of concentration.

### Host-seeking ability of *Aedes albopictus* exposed to chemical compounds

The effect of different concentrations of chemical compounds on the response to human odor are showed in Table 2. Except for anisaldehyde, all compounds at different concentrations inhibited the ability of mosquitoes to respond to human odor to a certain degree, as the average capture rates at the treatment port were lower than the control. The inhibition effect was much more pronounced at the highest concentration (96%), indicated by capture rates higher than 31.2% (the average capture rate to human odor under the same condition) at the control port. At 6%, only citral showed a good inhibition effect (capture rate of 6.5% at treatment port vs. 38.2% at control port). A positive relationship was observed between the inhibition effect and the concentration of all compounds except anisaldehyde (Figure 3). Anisaldehyde at lower concentrations (6–48%) enhanced the response to human odor, as indicated by negative relative inhibition rates.

**Table 3.**
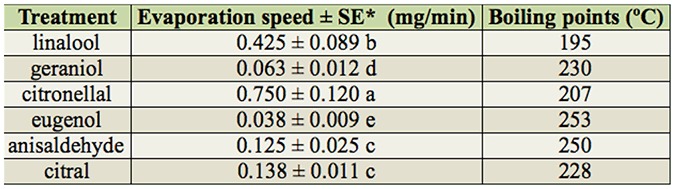
The speed of evaporation of compounds.

### Evaporation rate

The evaporation rates of the tested compounds were significantly different (Table 3). Citronellal had the highest evaporation rate, followed by linalool. Eugenol had the lowest evaporation rate, followed by geraniol. The evaporation rates of citral and anisaldehyde were in the middle and were not significantly different from each other.

## Discussion

In general, insect's odor-mediated behaviors depend on sensory input resulting from multiple chemical receptors that send different signals to the brain. A given chemical cue induces a certain behavioral response by activating some receptors and inhibiting or having no effect on others. Chemical ecological studies have indicated that higher concentrations of a chemical cue are often required for insects to exhibit repellent behavior (moving away from the source) because the responsible receptors have a higher response threshold. As a result, chemicals may act as attractants to insects at lower concentrations but as repellents at higher concentrations, and this observation is often assumed as a general framework in insect chemical communications (Finch 1978). Our study confirmed that the concentration of a chemical does effect the behavioral response, and also showed that the assumed general framework is inappropriate in many cases.

Among the tested chemicals, only citronellal exhibited this typical observation. The evaporation rate seems to be an influential factor, as citronellal had the highest evaporation rate compared to the other compounds. However, compounds with similar evaporation rates, e.g. anisaldehyde and citral, induced very different dose-dependent behavioral responses. The results indicate that the behavioral response of mosquitoes to chemical cues is complex and the underlining mechanism is poorly understood (Davis 1985). Future studies will focus on using biochemical and molecular tools to understand and explain the results observed in this study.

Mosquitoes locate their blood hosts by responding to chemical cues emitted by the hosts. It is not surprising to observe different behavioral response toward a chemical alone vs. in the presence of a host, as observed in this study. It is important to characterize the effect of host presence on mosquito behavioral response to a given chemical so that this knowledge can be applied in mosquito management. For example, citral, geraniol, and linalool are suitable for the development of spatial repellents, as they inhibited mosquitoes' host-seeking ability and provided good host protection. For citronellal, a high concentration (96%) is required for it to have a spatial repellent effect and provide host protection. If concentration is below 12%, citronellal has an attractant effect and provides no protection to human hosts. When eugenol was tested alone, no repellent effect was detected at any concentration. However, all tested concentrations of eugenol showed relatively strong host-seeking disruption and provided good host protection, making it a good candidate for spatial repellent. Anisaldehyde should be cautiously considered for development as a spatial repellent because it attracts mosquitoes at lower concentrations and a high concentration (96%) is required to provide meaningful host protection.

These results demonstrated the importance of this type of study, as it provides practical information to guide formulation and releasing technology in developing safe and effective anti-mosquito products.
